# Strengthening caesarean birth: Sub-Saharan Africa health system evaluation: Scoping review

**DOI:** 10.4102/phcfm.v16i1.4128

**Published:** 2024-04-29

**Authors:** Patrick Minani, Andrew Ross

**Affiliations:** 1 Department of Public Health Medicine, Faculty of Health Sciences, University of KwaZulu-Natal, Durban, South Africa; 2 Department of Family Medicine, Faculty of Health Sciences, University of KwaZulu-Natal, Durban, South Africa

**Keywords:** health system, caesarean birth, Sub-Saharan Africa, public health, skilled birth attendants, anaesthetic safety

## Abstract

**Background:**

Promoting safe caesarean birth (CB) is a challenge in sub-Saharan Africa (SSA) where maternal and neonatal mortality rates are high due to inadequate maternal health services. Although the CB rate in SSA is lower than the World Health Organization (WHO) recommendation, it is often associated with high maternal and neonatal mortality.

**Aim:**

The aim of this scoping review was to report on the extent to which SSA health systems deliver safe CB.

**Methods:**

A systematic search across various databases identified 53 relevant studies, comprising 30 quantitative, 10 qualitative and 16 mixed methods studies.

**Results:**

These studies focused on clinical protocols, training, availability, accreditation, staff credentialing, hospital supervision, support infrastructure, risk factors, surgical interventions and complications related to maternal mortality and stillbirth. CB rates in SSA varied significantly, ranging from less than 1% to a high rate of 29.7%. Both very low as well as high rates contributed to significant maternal and neonatal morbidity. Factors influencing maternal and perinatal mortality include poor referral systems, inadequate healthcare facilities, poor quality of CBs, inequalities in access to maternity care and affordable CB intervention.

**Conclusion:**

The inadequate distribution of healthcare facilities, and limited access to emergency obstetric care impacted the quality of CBs. Early access to quality maternity services with skilled providers is recommended to improve CB safety.

**Contributions:**

This scoping review contributes to the body of knowledge motivating for the prioritization of maternal service across SSA.

## Introduction

Caesarean birth (CB) is a vital surgical procedure needed to manage some complications related to pregnancy and childbirth.^1^ Caesarean birth is an operative practice in which an incision is made through a mother’s abdomen and uterus to extract one or multiple babies, or infrequently, extract a dead fetus.^2^ Because of the perceived safeness of the procedure and the convenience (for the mother and doctor) of being able to plan birth, CB has become a popular mode of giving birth.^3^ As a result, the rate of CB has been rising in many countries around the world despite the World Health Organization (WHO) recommending that the rate of CB should not exceed 10% – 15% of deliveries.^3^

According to the WHO, between 1990 and 2014, the mean rate of CB ranged from 6% to 12.4% in low-resourced countries and 27% in developed countries^4^ with this rate increasing by 4.4% annually.^5^ When compared to continents across the world, Africa has the lowest rate of CB at 7.3%, Asia 19.2%, Europe 25%, Oceania 31.1%, North America 32.3%, with Latin America and the Caribbean reporting the highest rates of CB at 40.5%.^4,5^ These numbers are far from the 10% – 15% CB rate as recommended by the WHO. What is more, CB carries a significant risk of maternal and perinatal morbidity and mortality when compared with vaginal birth.^6,7^

Multiple maternal and perinatal risk factors have been associated with CB leading to increased morbidity and mortality^8^ when compared to vaginal birth. This is especially true in settings where there is a shortage of skilled staff, where there are insufficient medical facilities and equipment and where CBs are performed in less-than-ideal conditions.^1^ During CB, the mother has an increased risk of developing an intraoperative injury; post CB, the mother might develop bleeding or infection that could require a hysterectomy or could lead to death, and in the long term, the mother has an increased risk of developing pelvic adhesions. In subsequent pregnancies, the mother is at a greater risk of abnormal placentation, stillbirth, preterm birth at any stage of the pregnancy and uterine rupture.^9^

In sub-Saharan Africa (SSA), a CB is usually an emergency procedure that is done too often and has led to unnecessary negative outcomes on maternal and foetal health.^10^ In contrast, when a CB is done for the correct indications, this leads to reduced risk to the mother and newborn baby.^1^ The purpose of this scoping review was to look at the evidence that effective healthcare systems contribute to safe CB practices in SSA. The specific objectives were to determine the factors of health systems that influence CB outcomes, to assess and understand how health systems contribute to effective CB practice in SSA and to understand how these influence the indications for CB as well as the maternal outcome post CB.

## Methods

### Search strategy

A systematic search of primary studies in peer-reviewed journals was done on MEDLINE, ScienceDirect, JSTOR Health & General Sciences Collection, CINAHL with Full Text, Health Source: Nursing/Academic Edition, MEDLINE with Full Text, PubMed, Google Scholar, Cochrane Library, African Index Medicus (AIM), Bioline International, BioMed and EBSCOhost. The search was carried out using the following keywords with Boolean terms: Cesarean birth OR Cesarean section OR C-section AND factors OR determinant OR indications AND maternal mortality, OR morbidity AND maternal referral AND health system AND SSA. Articles that could not be accessed through the electronic search were sought through the University library. Articles retrieved from the searches were screened by the author, and references were managed using Endnote version 9. The review included research articles from January 2005 to October 2020, and the search was conducted in December 2020. Articles were initially screened using titles followed by further screening using abstracts of selected articles for eligibility (Risma). Articles that did not meet the eligibility criteria at the title and abstract screening were excluded and only those that fulfilled the inclusion criteria were considered for full article review. Studies were selected based on the following criteria:

- Only studies published in English were considered.
- Only articles focusing on CB in SSA published between 2005 and 2020.
- Studies that evaluated the frequency and accessibility of CB.
- Studies that included the maternal and perinatal outcomes after undergoing CB.
- Studies considered were cross-sectional, retrospective, descriptive, analytical and those that used mixed methodology.
- Studies that considered health system factors (training, support, accreditation or licensing of operators, protocols, equipment, staffing) that influenced CB outcomes and complications associated with CB.

#### Primary outcomes

The outcomes of interest included the following: health system factors that influenced the practice of CB in both rural and urban areas of SSA. These included the use of clinical protocols, training of doctors, availability of anaesthetists and nurses accredited in maternity services, credentialing of qualified staff, the occurrence of review or supervision within the hospital and support or infrastructure.^8,11^ The other outcomes were to identify and describe the indications associated with the pregnancy, the surgical intervention, perioperative complications associated with maternal mortality and stillbirth. Other outcomes of interest included the country of study, risks associated with CBs such as stillbirths, bladder injury, etc.

### Data extraction process

Data extraction from the included studies was carried out by the author under his supervisor’s guidance. Data extraction was done using content analysis. Articles were screened to identify the outcomes of interests and these were entered into a data extraction table. The data extraction form was generated from Microsoft Word and drawn up based on the study variables and types of data to be collected. The following study characteristics were extracted: publication characteristics (authorship list, date of publication, journal of publication and country or language of publication), study design information and outcomes assessed.

Narrative synthesis was used to synthesise the data, as most of the outcomes were qualitative in nature such as identifying risk factors, barriers and facilitators in health systems and facilities. Narrative synthesis enabled the researcher to describe and explain issues, which were discussed in the screened articles.

### Study selection

**FIGURE 1:**
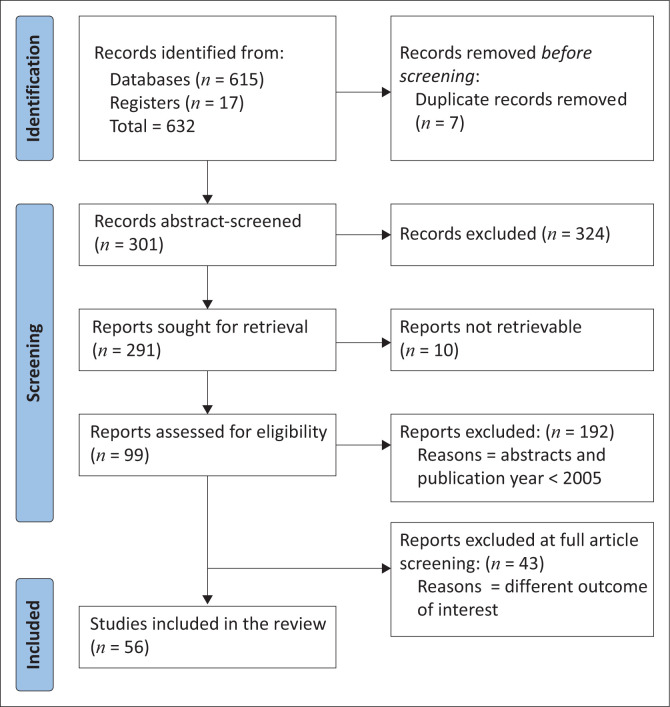
PRISMA flow diagram of the analysis procedure.

The data were obtained after searching on PubMed and Google Scholar in English using the keywords. A flow diagram documenting the process of study selection is in Figure 1. A total of 625 titles were identified through the literature search, other sources and the removal of duplicates. Evaluation of these citations by the author excluded a total of 324 articles because of not meeting the inclusion criteria, leading to 301 articles for review in full text. Among these articles, a total of 245 were excluded by the reviewer (reasons provided in Figure 1), leaving a total of 56 publications for final inclusion. PRISMA provides an overview of the primary features of these.

### Ethical considerations

Ethical clearance to conduct this study was obtained from the University of KwaZulu-Natal Biomedical Research Ethics Committee (No. BREC/00002286/2021).

## Results

### Characteristics of included studies

**FIGURE 2:**
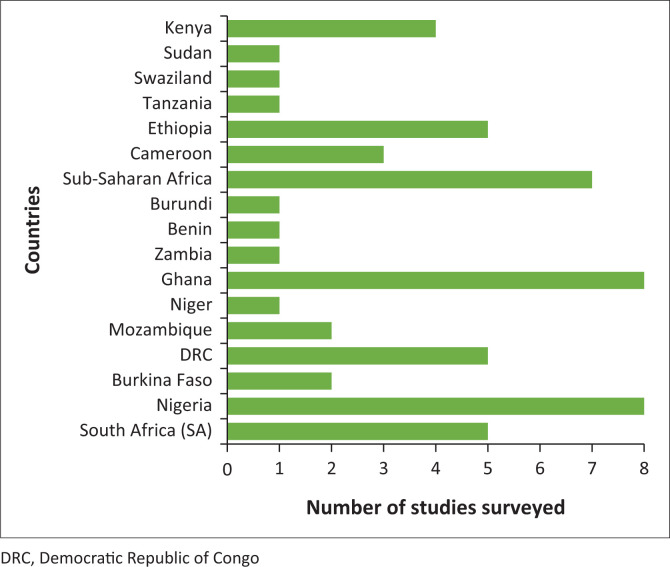
Distribution of surveyed studies according to first-author, country-institution affiliation (*N* = 56).

Figure 2 indicates the distribution of the 56 studies with respect to study sites where the studies were conducted.

Thirty of the studies were quantitative; 10 were qualitative and 16 used mixed methods. In terms of outcomes, 5 reported on the use of clinical protocols; 7 on the training of doctors, anaesthetist and nurses; 6 on the availability of anaesthetists and 4 on nurses accredited in maternity services, credentialing of qualified staff; 4 on the occurrence of review or supervision within the hospital and 12 on support or infrastructure. In addition, 5 identified and described the risk factors associated with the pregnancy, 6 described surgical interventions and 7 described perioperative complications associated with maternal mortality and stillbirth. Further details can be seen in Table 1.

**TABLE 1:** Characteristics of included studies.

Article number	First author (publication year)	Reference	The country where the study was conducted	Outcome assessed	Study design
1	Give C. (2019)	12	Mozambique	Maternal healthcare	Qualitative
2	Dare (2019)	8	Multiple countries	Maternal healthcare	Qualitative
3	Olusanya (2009)	13	Nigeria	Maternal healthcare	Quantitative
4	Sawadogo (2019)	9	Burkina Faso	Maternal healthcare	Quantitative
5	Solanki (2020)	14	South Africa	Maternal healthcare	Qualitative
6	Michel (2019)	1	DR Congo	Maternal healthcare	Quantitative
7	Ajayi (2021)	15	Nigeria	Maternal healthcare	Mixed method
8	Taye (2021)	16	Ethiopia	Prevalence and factors associated with caesarean section	Quantitative
9	Henri	6	Cameroon	Maternal healthcare	Qualitative
10	Richard	17	Burkina Faso	Maternal healthcare	Qualitative
11	Asuquo (2016)	2	Nigeria	ANC and childbirth	Mixed method
12	Harrison (2016)	5	South Africa	Maternal healthcare	Mixed method
13	Atombosoba (2015)	11	Nigeria	Maternal healthcare	Qualitative
14	Dare (2019)	8	SSA countries	Maternal healthcare	Quantitative
15	Igwebueze (2015)	18	Nigeria	Maternal healthcare	Mixed method
16	Seidu (2020)	19	Ghana	Maternal healthcare	Quantitative
17	Udoye (2016)	20	Nigeria	ANC and childbirth	Quantitative
18	Egbe (2016)	21	Niger	Maternal healthcare	Mixed method
19	Echoka (2014)	22	Kenya	Maternal healthcare	Qualitative
20	Chiabi (2014)	23	Cameroon	ANC and postnatal care (PNC)	Quantitative
21	Dankwa (2019)	24	Ghana	Health determinants and maternal healthcare	Quantitative
22	Betrán (2016)	4	Multi-Country	Maternal healthcare	Quantitative
23	Ndungo (2019)	25	DR Congo	ANC and childbirth	Mixed method
24	Enabudoso and Isara (2011)	26	Nigeria	Prevalence of satisfaction and associated factors, among parturient women who had recently delivered by caesarean	Mixed method
25	Ali (2012)	27	Sudan	ANC and health determinants	Quantitative
26	Adu-Bonsaffoh (2014)	28	Ghana	Maternal healthcare	Quantitative
27	Massyn (2017)	29	South Africa	Maternal healthcare	Quantitative
28	Belay (2014)	30	Ethiopia	ANC and childbirth	Mixed method
29	Foumane (2014)	31	Cameroon	Maternal healthcare	Quantitative
30	Kinenkinda (2017)	10	DR Congo	Maternal healthcare	Mixed method
31	Fetene et al. 2022	32	Ethiopia	ANC and childbirth	Quantitative
32	Kampo (2019)	33	Ghana	ANC and childbirth	Quantitative
33	Omondi (2017)	34	Kenya	Maternal healthcare – patients’ pre- and postoperative anxiety levels and demographics	Quantitative
34	Monticelli (2012)	35	South Africa	Differences in care that pregnant women receive in different parts of the country and to illustrate where inequity of resource allocation is occurring	Mixed methods
35	Benwu et al. 2019	36	Ethiopia	Maternal healthcare – knowledge and skill towards cardiopulmonary resuscitation	Quantitative
36	Belay (2014)	30	Ethiopia	Maternal healthcare	Mixed method
37	Maine D (2009)	37	Swaziland	Maternal and child health	Mixed method
38	Gabrysch S. (2011)	38	Zambia	ANC and childbirth	Mixed method
39	Yaya S. (2020)	39	Burundi	Maternal healthcare	Qualitative
40	Sharma G. (2015)	40	Multi-countries	Maternal healthcare	Mixed method
41	Vora K. S. (2019)	41	Multi-countries	Maternal healthcare	Mixed method
42	Plus F. C. (2019)	42	Multi-countries	Maternal healthcare	Mixed method
43	Gyaase et al. (2023)	43	Ghana	Prevalence and factors influencing caesarean section (CS) deliveries	Quantitative
44	Manyeh et al. (2018)	44	Ghana	Rate of CS	Quantitative
45	Kinenkinda et al. (2017)	10	DR Congo	The frequency, the indications and the maternal and perinatal morbidity and mortality in patients undergoing CS in Lubumbashi	Quantitative
46	Belay et al. (2022)	45	Ethiopia	Maternal and perinatal outcomes of caesarean birth (C/D) performed in the second stage of labour compared with the first stage in the Ethiopian setting	Quantitative
47	Monticelli (2012)	35	South Africa	C-section rates in public sector hospitals	Quantitative
48	Long et al. (2015)	46	Mozambique	Explore changes in CS rates between 1995 and 2011 by area, place of delivery and maternal socioeconomic factors	Quantitative
49	Kabongo et al. (2017)	3	DR Congo	The coverage of caesarean needs and quality of services in maternity wards	Quantitative
50	Ugwu N. (2015)	47	Nigeria	Sociocultural factors that reinforce delays and nonacceptance of CS	Mixed methods
51	Solomon (2019)	48	Ethiopia	Prevalence of CS and associated factors in University of Gondar Comprehensive Referral Hospital, North West Ethiopia, Gonda	Quantitative
52	Ganle et al. (2014b)	49	Ghana	Maternal CS access	Quantitative
53	Ochieng et al. 2020	50	Kenya	Caesarean birth socioeconomic patterns and neonatal survival outcome	Quantitative
54	Juma et al.2017	51	Kenya	Factors associated with CS	Quantitative
55	Ravit et al. (2018)	52	Benin	Change in CS prevalence	Quantitative
56	Bishop et al. (2019c)	53	Multi-country	In-hospital maternal mortality and complications	Quantitative
57	Litorp et al. 2013	54	Tanzania	Trends in CS rates and outcomes among a variety of obstetric groups	-

Note: Please see the full reference list of the article, Minani P, Ross A. Strengthening caesarean birth: Sub-Saharan Africa health system evaluation: Scoping review. Afr J Prm Health Care Fam Med. 2024;16(1), a4128. https://doi.org/10.4102/phcfm.v16i1.4128, for more information.

ANC, antenatal care; SSA, sub-Saharan Africa.

**TABLE 2:** Key health system issues identified.

First author (year of publication)	Reference(s)	Focus of study	Health systems issues identified
Give (2019), Michel (2019)	1, 12	Determinants and factors influencing the prevalence of uterine rupture in a tertiary rural hospital in the Niger Delta	Inadequate referral pathways Inadequate training
Ajayi (2020); Olusanya (2009); Michel (2019); Solanke (2009)	1, 13, 15, 47	Studies conducted in Lagos, Nigeria, identified a lack of skilled birth attendants in most Nigerian rural areas, compared to studies conducted in urban areas in Lagos, DR Congo, Douala, Cameroon and Ghana which identified the unavailability of qualified staff.	Lack of skilled birth attendance (credentials)
Henri (2019)	6	A comparative study on first-stage versus second-stage caesarean section on maternal and perinatal outcomes (Open Journal of Obstetrics and Gynecology)	Inconsistent use of protocols
Sawadogo (2019)	17, 40	This multi-country analysis study conducted in Burkina Faso concluded that clinical guidelines are not updated and not used in maternity service. Malawi is one of the types of evidence compared to another study conducted in the same country where the protocol was used by nurses in the postoperative ward to manage postoperative cases.	Inconsistent use of protocols
Atombosoba et al. (2015)	3, 11, 12, 22, 49	Studies conducted in Kenya, Ghana, DR Congo and the Niger Delta reported poor accessibility to health facilities because of poor infrastructures, including poor roads, health facilities and adequate ambulances. These elements contributed to a delay in reaching facilities.	Inadequate infrastructures and insufficient health facilities
Henri (2019)	6	Studies conducted in Yaoundé Cameroon reported a shortage of staff at the EmOC. A single qualified anaesthetist for all surgical emergencies.	Insufficient qualified staff (lack of staff with credentials)
Bayou (2016)	57	A study conducted in Addis Ababa, Ethiopia, reported the availability of advanced obstetric services in Addis Ababa, Ethiopia.	Insufficient qualified staff
Seidu (2020)	19	This study showed that maternal age increased the risk of complications during CB.	Lack of skilled staff
Solomon (2019)	48	This study showed that women who got pregnant two to four times contributed to the proportion of CB rate of 29.7% at Gondar University Hospital, Ethiopia.	Poor infrastructure
Ugwu (2015)	47	This study showed that emergency CB accounted for more than 90% of all CB and was associated with the delay in access to health services.	Inadequate health facilities

Note: Please see the full reference list of the article, Minani P, Ross A. Strengthening caesarean birth: Sub-Saharan Africa health system evaluation: Scoping review. Afr J Prm Health Care Fam Med. 2024;16(1), a4128. https://doi.org/10.4102/phcfm.v16i1.4128, for more information.

CB, caesarean birth.

### Health system factors influencing caesarean birth outcome

The main risk factors that influenced maternal and perinatal mortality and morbidity after CB included poor referral and counter-referral systems, inadequate healthcare facilities, poor quality of CBs or lack of skilled staff, inequalities of access to maternity care and emergency obstetric care (EmOC) and affordable CB intervention.^1,3,9,11,15,18,55^

An international, prospective, observational cohort study conducted in Africa on CB found that in many countries common complications that led to maternal deaths were intraoperative and postoperative bleeding, reported in 136 (3.8%) of 3612 mothers in the study; 14 (2%) of 626 patients had postoperative complications that were associated with in-hospital deaths.^8,55^ However, the major obstetrical risk factors related to CB were infectious complications, operative and postoperative bleeding and stillbirth,^1,3,9,11^ as well as complications associated with both general and high spinal anaesthetists (a composition of failed intubation, aspiration, cardiac arrest and hypoxia).^8^ A retrospective study of hospital records conducted in four hospitals between 2013 and 2016 in Goma, Northeast of the Democratic Republic of Congo, reported 4534 deliveries including 736 (16.2%) by CB. This study showed that fistulae were the most common outcomes because of prolonged labour. Another study conducted in Ouagadougou, Burkina Faso, in 2018 revealed that the delay in referring emergency obstetric cases contributed to 47.06% of maternal mortality.^9^ Recommendations from these studies highlighted that promoting access to CB practice and providing a quality surgical operation for all CD indications (elective or emergency cases) as well as limiting CB overuse could avert and/or reduce much of the maternal death associated with CB.

### Accessibility and capability of sub-Saharan African countries to perform caesarean birth

In many SSA countries, the healthcare systems are characterised by insufficient healthcare facilities, finances and inadequate resources.^5^ Access to maternal health services remains a challenge in SSA, even though some countries have adopted a user-free maternal healthcare policy.^42,55,56^ A study conducted in Nigeria and Ghana revealed that inequality in access to CB intervention is still present despite the user-free maternal healthcare policy^15^ and that these inequalities exist among women of different sociodemographics.^15^ A similar study conducted in Ghana showed that the wealth status of pregnant women influenced the rise of the CD rate to 27.5% when compared to the percentage of poor women of whom only 5% had CB.^15^ However, living in a rural area and a low level of education increased the challenge of accessing a CB because of inadequate health infrastructures and a shortage of staff.^15^ Such studies highlight the fact that free access to maternal services cannot resolve the inequality regarding access to CB intervention. It is therefore important to improve the quality of the service, especially maternal healthcare services with access to skilled providers and skilled attendants, and sustain access to health services that reduce the maternal mortality associated with CB intervention.^15^

A study conducted in South Africa discussed characteristics of facility-based CB rates and safety in the public sector and emphasised the inequalities between the provinces. These included^1^: (1) the necessity of access to a safe CB intervention throughout the entire country and (2) the process to avert the rising rates of CB as this does not confer a benefit to maternal health.^14^ In addition, this study highlighted the need to improve the health system in the public sector, specifically the quality of the service delivery (skilled doctors, nurses and anaesthetists that can ensure a safe CB and manage complications related to CB) and create an environment for a safe CB in terms of equipment, infrastructure and quality care to be able to manage the complications related to CB. In ddition, the report highlighted that a strategy is needed to: (1) ensure patients are adequately evaluated before booking for CB, (2) ensure a reduction in primary CB, (3) ensure protocols that will guide the provision of vaginal births after CB and (4) encourage vaginal births in selected cases, the implementation of which may reduce the increasing rate of CB and complications related to CB.^18^

### Caesarean birth prevalence

Studies conducted in Cameroon and Nigeria reported a facility-based CB rate of 29.4% and 29.6%, respectively, which is much higher than the 10% – 15% WHO recommended rate and indicates that there is overuse of CB in the studied populations^6,18^ and higher than the average CB rate (20%) in other developing countries.^34^ Furthermore, a high proportion of the cost of CB is related to real healthcare costs, which can bring a significant burden on the health system.^44,54^

Table 3 shows the CB frequency in SSA where the CB frequency varies from 0.26% to 29.7%.^6,18^ The highest facility-based CB rate in this scoping review was 29.7% at Gondar University Hospital, Ethiopia and the lowest was a rate of 0.28% in Burkina Faso.^9,48^ The high rate of 29.7% did not confer any benefit to reducing maternal and perinatal mortality and was significantly higher than the recommendation from the WHO, which recommends the CB rates of the country should not be greater than 10% – 15%.^27^ However, a rate of 0.28% is also of concern and contributes to significant maternal morbidity (in the form of fistulae) and neonatal morbidity and mortality.

**TABLE 3:** Caesarean birth rate (%).

First author (year of publication)	Reference	Caesarean birth rates (%)	Comments
Ajayi (2021)	15	6.1	The prevalence of CB shows that inequality in access to CB persists despite the free maternal healthcare policy in Nigeria.
Dikete Ekanga	1	16.2	This study conducted in DR Congo reported that a CB rate of 16.2% is associated with dystocia (difficult and protracted labour), scarred uterus and foetal suffering.
Dankwa (2019)	24	11.4	This study revealed that in Ghana, women with wealthy status contributed to an increase in the CB rate by 11.4%.
Henri (2017)	6	29.6	This study conducted in Douala, Cameroon, shows that mechanical dystocia, fresh uterine scar and acute foetal distress (AFD) contributed to a high CB rate of 29.6%.
Igwebueze (2015)	18	29.6	This study conducted in Nigeria showed that a CB rate of 29.6% is associated with eclampsia, uterine rupture, obstructed labour and puerperal sepsis leading to maternal mortality.
Kabongo (2017)	3	21.1	This high rate of CB was associated with pregnancy complications and elective CB in both the health facilities of Kansasa and Tshilenge (in the same province of DR Congo).
Olusanya (2009)	13	Elective 9.6 Emergency 34.4%	This study shows that parity, lack of antenatal care and prolonged or obstructed labour were associated with an increased risk of emergency CB compared with elective CB in Nigeria.
Richard (2008)	17	1.9 in 2003 24.5 in 2006	A study conducted in Ouagadougou, Burkina Faso, reported that the facility-based rates of CB increased each year. However, this increase happened without an increase in maternal and perinatal post-caesarean mortality.
Sawadogo (2019)	9	0.28	This study conducted in Burkina Faso focusing on maternal death reported a facility-based rate CB of 0.28%. However, this rate was beneficial to maternal health and the recommendation of the WHO.
Seidu (2020)	19	18.5	This study showed that there is evidence that advanced maternal age increases the risk of giving birth to CB.
Ugwu (2015)	47	14	This study showed that emergency CB accounted for more than 90% of all CB and was associated with the delay in access to health services.
Solomon (2019)	48	29.7	This study showed that women who got pregnant two to four times contributed to the proportion of CB rate of 29.7% at Gondar University Hospital, Ethiopia.
Yaya (2020)	39	16	The disparity in CB was present in both surveys, and this increased over time. Optimal rates do not indicate why some have easy access to CB, while others who may need it do not.

Note: Please see the full reference list of the article, Minani P, Ross A. Strengthening caesarean birth: Sub-Saharan Africa health system evaluation: Scoping review. Afr J Prm Health Care Fam Med. 2024;16(1), a4128. https://doi.org/10.4102/phcfm.v16i1.4128, for more information.

CB, caesarean birth; WHO, World Health Organization.

In SSA, the low CB rate has contributed to the rise of foetomaternal mortality and morbidity.^27^ A facility-based study conducted in Nigeria in 2020 demonstrated a CB rate of 6.1%, which the authors concluded was because of the inequality of access to and use of maternal health intervention for the majority of women living in the rural area, which occurred despite the provision of free access to the maternal healthcare in the study setting.^27^ Factors that contributed to the low CB rate were inadequate health facilities, poor referral, counter-referral systems and substandard equipment resulting in adverse outcomes despite the adoption of a user-free maternal health services policy in Nigeria.^27^ Challenges of managing emergencies and referral cases for CB are dominant issues in several studies and highlight the health systems challenges in CB management.^1,3,28^

An analysis of maternal deaths in the context of CS in South Africa discovered that the risk of a woman dying after CS was nearly three times that of maternal mortality following vaginal delivery (VD).^5^ The study reported that haemorrhage was the primary cause of death (5.5 deaths per 10 000 CS administered); embolism was 4.5 times more frequent after CB than VD and hypovolemic shock was 4.8 times more likely and that the number of deaths per 10 000 CB cases ranged from 10.1 to 31.9.^5^ When the CC rate in a facility was compared to the case fatality rate, a negative link was discovered, indicating that in places with a lower CB rate, the case fatality rate was higher.

According to the 2015/16 District Health Barometer, the CB rates in South Africa’s public sector facilities climbed from 15.1% in 2006 to 24.1% in 2015. According to Solanki et al.,^14^ Gauteng and the North-Western Cape had the biggest increases. The availability of regional, provincial tertiary and national central hospitals and the unequal access to CB intervention may be the reasons for the notable variation in CB rates among provinces. Additionally, the high concentration of community health centres (CHCs) in predominantly rural provinces may have contributed to their lower proportion.

Even though CB is an effective procedure to avert maternal and perinatal mortality when used appropriately, it is not without risk that includes both immediate and long-term complications.^50^ In SSA countries, when emergency CBs are performed, there is often a high risk of complications for both the mother and the newborn. Early access to maternity services, rapid diagnosis, deciding to refer a patient after failure to induce labour with misoprostol and the early decision to refer the pregnant woman to another hospital that can safely perform a CB have been shown to decrease those risks.^30^ However, it is important to assess the pregnancy in terms of detecting potential factors (by attending antenatal care [ANC] and early ultrasound) that can help to avert complications related to CB. In this study, some causes of those risks were analysed including the poor maternal and newborn outcome, which was the result of lack of quality antenatal care and prenatal care, insufficient numbers of skilled health staff in the maternity unit, inadequate placement of healthcare facilities and difficulties to access to referral facilities.^10,31,58^ This study highlighted the need to prepare strategies that will empower the health systems to reduce maternal and perinatal mortality because of CB practice in SSA. This is particularly important for rural and overloaded DHs where there is a significant shortage of qualified staff and resources to provide a safe CB.

Reforms in the health systems and specific coaching of the staff in the maternity services (medical officers, gynaecologists, anaesthetists and midwives) are essential to decrease these complications and uncertain outcomes for pregnant women and babies after undergoing CB. However, to achieve this, health systems should provide good-quality or comprehensive EmOC that should always be available and accessible, that every woman should give birth within a maternity unit staffed by a professional, skilled attendant and that these services should be included in health systems, as well as the provision of safe obstetric anaesthesia.^8^ Poor outcomes from CB in SSA are often caused by factors related to the health systems, including inadequate training of mid-level providers, such as clinical officers and assistant medical officers, poor use of national treatment protocols, staff who are not authorised to perform CB, poor patient records and a lack of regular review of the indications for CBs that were performed.^1^ The common health system factors that led to poor outcomes were the inadequate provision of resources that can save women in an obstetric emergency, absence or poorly skilled birth attendants, inappropriate distribution of health facilities and poor referral and counter-referral systems that limit access to EmOC.^38,59^ This lack of access to safe CBs could be the predominant reason why some SSA counties reported a facility-based CB rate of less than 5% rather than the 10% – 15% recommended by the WHO in public and private healthcare facilities.^39^

Health system factors that influenced the CB outcomes negatively were mainly identified in rural areas. However, some countries have shown the political will to improve their health system, for example, in Maputo, Mozambique training of health providers on the referral of a patient to another health facility or district hospital, and training of supervisors on supportive supervision was provided.^12^ Another study conducted in Douala, Cameroon, identified the accreditation of skilled obstetricians and the availability of a single anaesthetist for all surgical emergencies.^6^ Furthermore, a study conducted in Lagos, Nigeria, reported that 57% of pregnant women were attended by a skilled health professional.^6^ These factors show the potential for improvement of the health systems and the impact that this could have on improving CB outcomes within and among SSA countries.

### Indications for caesarean births

Table 4 shows the indications for CB in SSA. The main indications for CB reported by these authors were dystocia (difficult and protracted labour), scarred uterus, foetal distress, rupture or preuterine rupture, placenta previa, malpresentation, cephalopelvic disproportion (CPD), non-reassurance foetal heart and pregnancy-related hypertensive disorder, breech presentation and cord prolapse.^3,6,11,17,18,22,23,48^

**TABLE 4:** Indication for caesarean birth.

First author (year of publication)	Reference(s)	Indications for caesarean births	Comments
Atombosoba and Igwebueze (2015)	11, 18	Uterus rupture, prolonged labour and failure of misoprostol with or without a scar	Studies were conducted in Lagos, Nigeria and reported similar indications and pregnancy complications that were associated with maternal deaths.
A. Chiabi (2014)	23	Prolonged labour, membrane rupture >18H (OR: 3.87)	The CB proportion in this study conducted in Cameroon was increased by prolonged membrane rupture OR: 3.87
Dikete Ekanga (2019)	1	Dystocia (difficult and protracted labour), scarred uterus and foetal suffering	Additionally, a small proportion of women were bleeding. This influenced the indication of the CB to save both the mother and baby’s life.
E. Echoka (2014)	22	Obstructed labour, severe preeclampsia, abnormal presentation and obstructed labour	This study conducted in Malindi, Kenya, revealed that the delay in access to emergency obstetric care (EmOC) services increased complications before the CB procedure.
Richard and Henri (2008–2019)	6, 17	Acute foetal distress, placenta praevia, severe preeclampsia, retroplacental haemorrhage, uterus rupture, malpresentation, fetopelvic and disproportion	Studies conducted in Cameroon and Burkina Faso reported a high rate of referral cases at EmOC service (46.8%) and recorded a CB rate of 29.06%. These referral cases increased the CB rate and complications related to the intervention.
Egbe and Sawadogo (2016–2019)	9, 21	Puerperal infection and uterus rupture feverish fainting episodes	Studies conducted in Nigeria and Burkina Faso identified common indications for CB and post CB complications such as antepartum haemorrhage, stillbirth and infections, which contributed to maternal mortality (147.68 maternal deaths per 100 000 live births) in Burkina Faso.

Note: Please see the full reference list of the article, Minani P, Ross A. Strengthening caesarean birth: Sub-Saharan Africa health system evaluation: Scoping review. Afr J Prm Health Care Fam Med. 2024;16(1), a4128. https://doi.org/10.4102/phcfm.v16i1.4128, for more information.

CB, caesarean birth.

### Risks associated with caesarean birth practice

Table 5 shows the maternal and perinatal risk factors that lead to complications when performing CB. The main risk factors reported by these authors were chronic hypertension, cardiac disease, lung disease or other medical risk factors.^1,8^ Furthermore, a study conducted in Burkina Faso reported postpartum and antepartum haemorrhage, stillbirth and infection.^9^ The ratio of maternal mortality by direct obstetric cause was 147.38 maternal mortality per 100 000 live births with a mean of 6.8 maternal deaths annually.^8,11,21^ In addition, some incidents related to the procedure were also reported including bladder injury, intraoperative haemorrhages and the release of sutures.^1^

**TABLE 5:** The maternal risks associated with caesarean birth.

First author (year of publication)	Reference	Risks associated with caesarean births	Comments
Atombosoba (2015)	11	Hysterectomy (10.34%), bladder injury and repair	This study conducted in Niger Delta shows that uterine rupture incidence was related to both maternal morbidity and the use of misoprostol on the uterus with or without a scar.
D. E Michel (2019)	1	Stillbirths were 4.4%; maternal death accounted for 0.1% of CB.	The common postoperative risk of complications related to CB in four referral hospitals in Goma/DR Congo included infections and stillbirth.
Ganle (2014)	49	While 4% of women did not receive maternity care component at all, 34% of women received ANC only.	Women who do not have access to maternity services increased their chances of complications associated with CB practice in Ghana.
Henri (2019)	6	Intraoperative haemorrhages (1.2%), maternal death (0.4%), stillbirths (3.2%)	This study conducted at Laquintinie Hospital (Douala, Cameroon) shows that risks of complications were associated with the high rate of emergency (46.8%) and referral (23.9) cases for CB.
Kabongo (2017)	3	Infectious complications (59.2%), haemorrhagic complications (28.9%)	The commonest complications related to CB were suppurative infectious (59.2%) and dropping/release sutures (35.1%) in both studies setting Tshilenge and Kansasa Hospital in the Western province of DR Congo.
Sawadogo (2019)	9	Haemorrhage, hypertensive disorders post CB, infections, haemorrhage and infectious diseases	This study was conducted in Burkina Faso and showed that women were exposed to poor management of post CB complications.
Udoye Patrick (2016)	20	Hysterectomy, bladder injury, and repair tubal ligation, rupture of a previous scar	These complications increased maternal mortality by 6.89% and morbidity in a tertiary rural hospital in the Niger Delta according to the author.

Note: Please see the full reference list of the article, Minani P, Ross A. Strengthening caesarean birth: Sub-Saharan Africa health system evaluation: Scoping review. Afr J Prm Health Care Fam Med. 2024;16(1), a4128. https://doi.org/10.4102/phcfm.v16i1.4128, for more information.

ANC, antenatal care; CB, caesarean birth.

## Discussion

This scoping review examined the role of healthcare systems in SSA, focusing on health system factors influencing CBs and maternal outcomes. Maternal care in SSA faces challenges because of factors such as inadequate facilities, lack of skilled staff and inequalities in access. Affordable interventions can help reduce CB complications, including intraoperative and postoperative bleeding, which contribute to maternal deaths. Promoting access to maternity care and limiting CB overuse could help reduce maternal mortality. Insufficient healthcare facilities in SSA countries, including Nigeria and Ghana, influencing maternal morbidity and mortality persist despite user-free maternal healthcare policies. Rural areas face greater challenges because of inadequate health infrastructures and staff shortages. Improving service quality, particularly with skilled providers, is crucial to reduce maternal mortality associated with CBs. To improve CB outcomes, the public health sector should enhance service delivery, including the provision of skilled doctors, nurses and anaesthetists, to ensure a safe and efficient CB management environment. Inadequate resources, lack of skilled birth attendants, inappropriate facility distribution and referral systems all contribute to poor health outcomes. To improve CB outcomes, health systems should ensure accessible, high-quality emergency care, professional maternity units and safe anaesthesia for every woman. Early access to maternity services, rapid diagnosis and referral to safe hospitals can decrease complications. Enhancing health systems, such as accreditation of skilled obstetricians and single anaesthetists, can also help.

### Caesarean birth prevalence

The study populations in Cameroon and Nigeria reported a facility-based CB rate higher than the recommended rate of 10% – 15%, indicating overuse and a high proportion of CB costs related to real healthcare costs.^5,6^ The facility-based CB rate in Ethiopia, with a rate of 29.7%, is significantly higher than WHO’s recommendation of 10% – 15%, contributing to significant maternal and perinatal morbidity and mortality and is also of concern.^7,8^ A study in South Africa found that maternal mortality after CB is nearly three times higher than after VD. Haemorrhage resulting in hypovolemic shock was the primary cause of maternal deaths.^9^ According to the 2015/16 District Health Barometer, CB rates in South Africa’s public sector facilities increased from 15.1% in 2006 to 24.1% in 2015, with the biggest increases in Gauteng and the North-Western Cape.^10^

### Health system factors influencing caesarean birth outcome

Emergency maternal healthcare is influenced by various factors, including inadequate healthcare facilities, lack of skilled staff, inequalities in access to maternity care and affordable intervention.^3,4,6,8,11,12,13^ Complications in maternal surgery, including intraoperative and postoperative bleeding, are common in many African countries, leading to maternal deaths.^12,14^ Complications in childbirth, including CB, are primarily linked to risk factors such as infectious diseases, stillbirth and spinal anaesthetist, which can lead to cardiac arrest and hypoxia.^3,8,11,13,14^ A study in Goma, Congo, found that prolonged labour led to vesicovaginal fistulae, and delayed EmOC contributed to 47.06% of maternal mortality.^8^ This study highlights that using guidelines to ensure appropriate indications, promoting access and providing quality surgical operations could reduce maternal death associated with CB.

### Contribution of health systems to effective caesarean birth practice in sub-Saharan Africa

Many SSA countries have inadequate healthcare systems because of insufficient facilities, finances and resources.^15^ Despite some countries adopting user-free maternal healthcare policies, access to maternal health services remains a significant challenge in SSA.^12,16,17^ A study in Nigeria and Ghana found that despite user-free maternal healthcare policies, inequality in access to CB intervention persisted among women of varying sociodemographic backgrounds.^4^ A Ghanian study revealed that the wealth status of pregnant women significantly influenced the increase in the CD rate to 27.5%, compared to a rate of only 5% among poor women.^4^

In rural areas, access to maternal services is challenging because of inadequate health infrastructures and staff shortages. Improving service quality, particularly with skilled providers, is crucial to reduce maternal mortality associated with CB intervention.^4^ The morbidity and mortality associated with CB in SSA highlights the need for increased safety measures and strategies to address the inequalities to ensure a comprehensive and effective CB service.^10^ There is also a need for strategies for early patient evaluation to reduce primary CB and encourage vaginal births, which would reduce the rate of CB and associated complications.^6^

The low CB rate in Nigeria, despite free access, has contributed to a rise in foetomaternal mortality and morbidity. This highlights the need to address factors such as inadequate health facilities, poor referral, counter-referral systems and substandard equipment, which contribute to the low CB rate, in addition to the adoption of user-free maternal health services.^18^ The management of emergencies and referral cases for CB is a major challenge in much of SSA, highlighting the health systems’ challenges in CB management.^1,11,19^

### Influence of health systems on the indications for caesarean birth as well as the maternal outcome post caesarean birth

This review identified an urgent need for strategies to reduce maternal and perinatal mortality because of CB practices in SSA, especially in rural areas with inadequate health systems plagued by a shortage of qualified staff and inadequate resources. Poor health outcomes in obstetric emergencies can be attributed to inadequate resources, lack of skilled birth attendants, inappropriate facility distribution and inadequate referral systems.^20,21^ Poor maternal outcomes following CB in SSA are results of inadequate training, poor use of national treatment protocols, unauthorised staff, poor patient records and lack of regular review of indications.^13^ Health systems should ensure accessible, high-quality EmOC, professional, skilled attendant maternity units and safe obstetric anaesthesia for every woman.^14^

The lack of access to safe CBs in some SSA counties contributes to a facility-based CB rate below the WHO’s recommended rate of 10% – 15%.^22^ Caesarean birth, when used appropriately, effectively prevents maternal and perinatal mortality, but it also poses risks from immediate and long-term complications.^23^ Early access to maternity services, rapid diagnosis and referral to safe hospitals can decrease complications.^24^ Many CB complications can be prevented by detecting potential risk factors during antenatal care and addressing issues such as quality care, availability of skilled health staff and adequate healthcare facilities.^25,26,27^

Caesarean birth outcomes could be improved by enhancing health systems, such as accreditation of skilled obstetricians and availability of single anaesthetists for surgical emergencies, as reported in studies in Cameroon and Nigeria.^5^ Some countries have shown the political will to improve maternal services, such as Mozambique’s training of health providers and supervisors on the importance of timely referrals and supportive supervision.^28^

### Strengths and weaknesses

The strength of this study is that it provides data from different types of studies evaluating health systems in SSA countries and their impact on the practice of CB. For example, a study conducted in a rural area in Nigeria reported that pregnant women travelled more than 20 km to attend a healthcare facility,^15^ while in Maputo, Mozambique, many training sessions were conducted with community health workers to ensure that they were clear about diseases or health concerns that need competent management, which included the referral of pregnant women for antenatal care and birth at healthcare facilities by a qualified personnel.^60^

Other studies conducted in an urban area of Lagos, Nigeria and Douala, Cameroon, found that only 57% of pregnant women were seen by qualified health staff with EmOC available and skilled birth attendants present at birth.^6,13,49^ Several health system points of quality and safety in low- to middle-resourced countries^41^ have been recognised, which include issues such as the absence of anaesthetic support, gaps in data quality (i.e. the name of an obstetrician who performed the CB and outcome of the procedure), insufficient equipment (this is associated with a delay to perform the procedure, anaesthetics complications and postoperative infections), gaps in training and supervision, lack of standardised protocols and skilled obstetricians as health system factors that influence the quality and safety of CB in many SSA countries.^42^ A study conducted in Ouagadougou, Burkina Faso, identified the availability of postoperative protocols (standing up early, removal of a urinary catheter and light meal on day 1) in the postoperative ward as contributing to better postoperative outcome, and additional training was provided to nurses for good implementation.^17^

The majority of SSA countries have a health workforce shortage, especially anaesthetists, and credentialing of the anaesthetist in the maternity unity was identified as an important health system activity to improve CB outcomes.^1,6,15,17,^ Studies conducted in Ouagadougou, Cameroon, identified that when doctors were trained in general and spinal anaesthesia to perform CD, CB outcomes improved.^9,17,40^

### Weaknesses of this scoping review

The weaknesses of this scoping review include the following:

- Language limitation – the study was limited to articles in English and therefore might have missed papers in other languages.
- Although the scope of the study was SSA, most papers were about specific countries that meant there were more papers from Nigeria, Ghana and Ethiopia and less from countries such as Zimbabwe, Zambia and Botswana.
- It is probable that using ‘AND’ on ‘maternal referral’ in the records search strategy constrained the study to those papers that explicitly covered maternal referrals. This may have resulted in the study focusing solely on maternal referrals in relation to CB, at the expense of other important studies about CB that did not directly cover maternal referrals.
- Only the main author was responsible for the data extraction. Although he worked under the guidance of his supervisor, this is a limiting factor.

## Conclusion

This scoping review has discussed health system factors that contribute to CB practices that have a significant impact on maternal and newborn outcomes. The data showed evidence of weakness in health systems in the majority of SSA countries, which impact on the implementation of CB practice. Effective reforms of the health systems are needed to ensure accessibility to maternal health services, quality EmOC, availability of skilled birth attendants at every delivery, and availability of staff and equipment to ensure safe CB when necessary. The training of staff and the use of protocols in the management of complications would also contribute to reducing maternal and neonatal mortality and morbidity. The rate of CB and the outcome of CB give a picture of the health system’s performance. It is difficult, however, to confirm whether the high proportion of CD in some SSA countries is associated with health system factors or inequity in access to the procedure. However, a good health system should provide a safe CB, be available and be accessible. The implementation of CB interventions needs to be performed in a secure environment with justifiable indications and with skilled staff (obstetrician, anaesthesist and nurse).
